# DNA damage and cytotoxicity in type II lung epithelial (A549) cell cultures after exposure to diesel exhaust and urban street particles

**DOI:** 10.1186/1743-8977-5-6

**Published:** 2008-04-08

**Authors:** Pernille Høgh Danielsen, Steffen Loft, Peter Møller

**Affiliations:** 1 Institute of Public Health, Department of Environmental Health, University of Copenhagen, Øster Farimagsgade 5, DK-1014 Copenhagen K, Denmark

## Abstract

**Background:**

Exposure to air pollution particles has been acknowledged to be associated with excess generation of oxidative damage to DNA in experimental model systems and humans. The use of standard reference material (SRM), such as SRM1650 and SRM2975, is advantageous because experiments can be reproduced independently, but exposure to such samples may not mimic the effects observed after exposure to authentic air pollution particles. This study was designed to compare the DNA oxidizing effects of authentic street particles with SRM1650 and SRM2975. The authentic street particles were collected at a traffic intensive road in Copenhagen, Denmark.

**Results:**

All of the particles generated strand breaks and oxidized purines in A549 lung epithelial cells in a dose-dependent manner and there were no overt differences in their potency. The exposures also yielded dose-dependent increase of cytotoxicity (as lactate dehydrogenase release) and reduced colony forming ability with slightly stronger cytotoxicity of SRM1650 than of the other particles. In contrast, only the authentic street particles were able to generate 8-oxo-7,8-dihydro-2'-deoxyguanosine (8-oxodG) in calf thymus DNA, which might be due to the much higher level of transition metals.

**Conclusion:**

Authentic street particles and SRMs differ in their ability to oxidize DNA in a cell-free environment, whereas cell culture experiments indicate that the particle preparations elicit a similar alteration of the level of DNA damage and small differences in cytotoxicity. Although it cannot be ruled out that SRMs and authentic street particles might elicit different effects in animal experimental models, this study indicates that on the cellular level, SRM1650 and SRM2975 are suitable surrogate samples for the study of authentic street particles.

## Background

The hazardous effects related to genotoxicity and cytotoxicity of particulate matter (PM) has been investigated in various cell culture experiments. Oxidative stress due to transition metals and particle-induced inflammation are believed to be important determinants for the generation of DNA damage such as strand breaks (SB) and oxidative damage to the DNA [1]. The 8-oxoguanine base lesion is generated in DNA during oxidative stress, and the 8-oxo-7,8-dihydro-2'-deoxyguanosine (8-oxodG) nucleotide is premutagenic if not repaired prior to DNA replication [2]. The level of oxidized guanine lesions can be measured by chromatographic techniques, antibody-based methods, and enzymatically recognized by e.g. the formamidopyrimidine DNA glycosylase (FPG) of *E. coli *[3,4]. There is evidence that exposure to urban air pollution increases the level of FPG sites in human mononuclear blood cells, whereas the level of SB is affected to a lesser degree [5-7].

The particulate fraction of air pollution in busy streets originates from traffic exhaust and especially from diesel powered vehicles as well as wear on breaks, tires and road material [8]. Humans are exposed to a mixture of air pollution particles, but in particular the diesel exhaust particles (DEP) have been extensively studied because of their chemical composition, small size, and large surface area. Samples of PM collected from urban air have different compositions and particle sizes, because of differences in location of sampling and collection time of the year. This makes it difficult to compare the detrimental effects of various samples of authentic particles. Moreover standard particle preparations might not mimic authentic air pollution particles. We have previously used the commercially available Standard Reference Materials (SRM) from the National Institute of Standards and Technology (NIST) to study effects of oxidative stress elicited by DEP in animal experiments and cell cultures [9-18]. The SRM1650 and SRM2975 preparations are samples of DEP that are well characterized for specific physical and chemical properties, such as concentrations of polycyclic aromatic hydrocarbons (PAH). Results from animal experimental models indicate that SRM1650 oxidizes DNA in the lung following pulmonary exposure [11,12,14]. In addition, oral administration of both SRM1650 and SRM2975 increased the level of oxidized DNA in the liver, lung and colon of rats [13,16,18,19]. However, it is important to note that the SRM1650 preparation has a higher content of PAH and iron ions compared to SRM2975 [20-22]. There is some indication from in vitro studies that SRM1650 generate more lipid-peroxidation than SRM2975 [22] and the inflammation potential may also differ between the SRMs [10]. This suggests that various types of model particles might display different responses in the same experimental setup, although the oral administration studies did not show differences in the DNA oxidizing potential between SRM1650 and SRM2975 [16,18,19].

The aim of this study was to investigate whether SRM1650 and SRM2975, which are commercially available DEP preparations, and authentic PM collected in a busy street (ASPM; authentic street particulate matter) have different oxidizing potential of DNA in a non-cellular environment and in cell cultures. To this end we investigated the induction of DNA damage in naked calf thymus DNA and A549 cells. The A549 cells originate from lung epithelial type II cells. These cells are thought to be progenitor cells for damaged type I epithelial cells in the alveoli. They are involved in the alveolar epithelial proliferative response observed by long-term inhalation of high doses of DEP and carbon black [23,24]. The cytotoxic effect of PM exposure was investigated as cell release of lactate dehydrogenase (LDH) and the ability to form colonies.

## Results

### Characterization of particles

Iron and copper were the most abundant transition metals of ASPM samples. The concentration of iron and copper in the ASPM samples were 37.4 ± 2.4 μg/mg and 2.1 ± .016 μg/mg, respectively (mean ± SD, n = 5). In comparison, there is much lower content of iron and copper in the SRM1650 (0.0031 and 0.020 μg/mg, respectively) and SRM2975 (0.0009 μg/mg and below limit of detection, respectively) [22]. It has not been possible to make a thorough assessment of the total concentration of PAH in the ASPM samples because of limited material. However, a recent collection of similar samples at the same location found that the concentration of 8 different PAH was 40 ng/mg [25]. The concentration of same PAH in the SRM1650 and SRM2975 preparations has been reported to be 47 and 29 ng/mg, respectively [20,21]. Collectively, the most pronounced difference between the SRMs and ASPM is the high concentration of transition metals in the latter.

### Cytotoxicity

**Figure 1:**
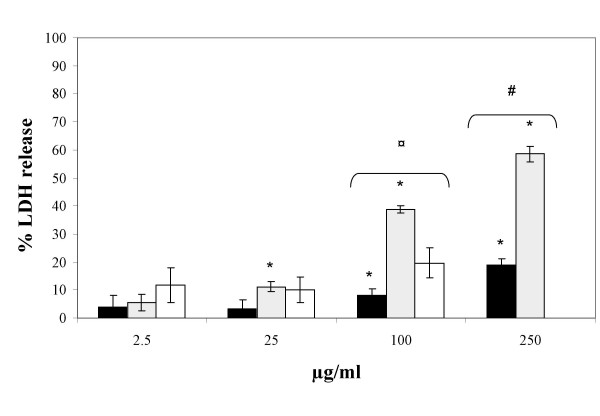
Cytotoxicity of A549 cells exposed to SRM2975 (solid bars), SRM1650 (hatched bars) and ASPM (open bars). The 100% maximum LDH release is obtained by treatment of cell cultures with Triton X-100, whereas the baseline LDH release in untreated cell cultures is 0%. Bars denote the mean ± SEM; (n = 3). * Statistically significant compared to control *p *< 0.05 (Kruskal-Wallis test). ^# ^Higher level of LDH release of SRM1650 compared to SRM2975 (*p *< 0.01, Mann-Whitney U test). ^¤ ^Higher level of LDH release of SRM1650 compared to SRM2975 and ASPM (*p *< 0.05, Kruskal-Wallis test).

The data on the cytotoxicity of PM, determined as release of LDH, are depicted in figure 1. The figure shows a dose-dependent increase in LDH enzyme activity in the cell culture supernatant at 25, 100 and 250 μg/ml for SRM1650 and at 100 and 250 μg/ml for SRM2975 (*p *< 0.05, ANOVA). The SRM1650 preparation was more cytotoxic to the cells than SRM2975 at 250 μg/ml (*p *< 0.01, Mann-Whitney U test) and more cytotoxic than both SRM2975 and ASPM at 25 μg/ml (*p *< 0.05, Kruskal-Wallis test). The ASPM preparation (all filters pooled) showed no significantly increased cytotoxicity at any doses compared to the control. The cytotoxicity at 250 μg/ml for ASPM was not measured. It should be noted that exposure to 100 μg/ml of ASPM was associated with approximately 20% increased LDH release that was not statistically significant at 5% level, but a similar level of LDH release was statistically significant for the SRM2975 samples. This suggests that the ASPM samples could be associated with cytotoxicity to a similar degree as the SRM2975 preparation.

Overall, the data from the LDH assay suggest that SRM1650 was more cytotoxic than SRM2975 at equal mass concentration and it cannot be ruled out that the ASPM preparations are slightly less cytotoxic.

### Colony forming ability

**Figure 2:**
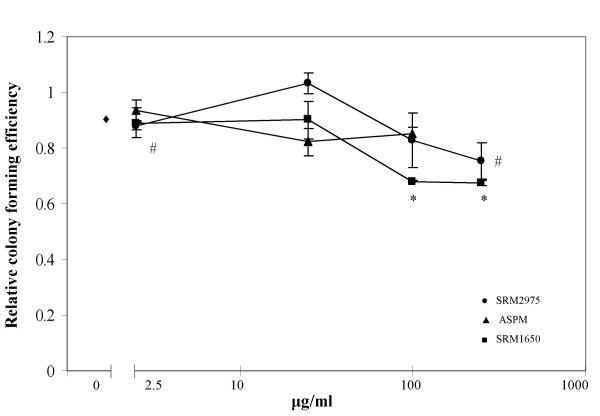
Colony forming ability expressed as relative to the number of colonies in the control, which are set to 1. The dots represents mean ± SEM; (n = 3). Decreased colony forming ability of cell cultures exposed to SRM2975 (^#^) SRM1650 (*) compared to the unexposed cultures (p < 0.05, Kruskal-Wallis test).

The results from the colony forming assay depicted in figure 2 demonstrated a moderate loss of ability to form colonies. For SRM1650, the loss was significant for doses at 100 and 250 μg/ml (*p *< 0.05, Kruskal-Wallis test). For SRM2975, the loss was significant for 2.5 and 250 μg/ml (*p *< 0.05, Kruskal-Wallis test). Considering the non-significant effects of SRM2975 at 25 and 100 μg/ml, the effect observed in cell cultures exposed at the dose of 2.5 μg/ml could be a chance finding. The ASPM preparation (all filters pooled) showed no significant loss of the ability to form colonies.

### In vitro generation of 8-oxodG in calf thymus DNA

**Figure 3:**
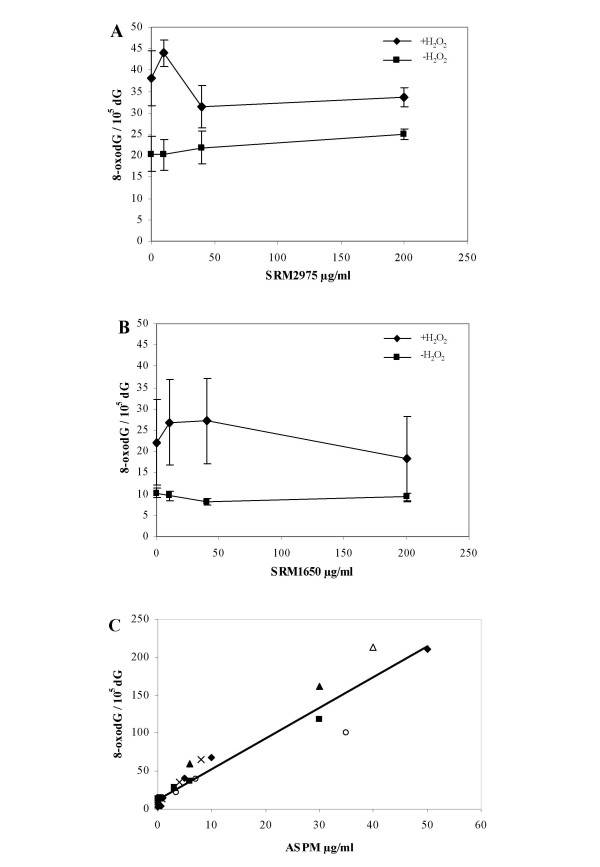
8-oxodG formation in calf thymus DNA measured with HPLC/ED after 30 minutes exposure to A) SRM2975 and B) SRM1650 and C) ASPM; ■ filter 1, ▲ filter 2, △ filter 3, ○ filter 4, ◆ filter 5. For SRM2975 and SRM1650 dots represents mean ± SEM; n = 3. The DNA was incubated at 37°C with 5 mM H_2_O_2_. For ASPM, the DNA was incubated at 37°C with 10 μM H_2_O_2_.

Figure 3 depicts the results of the generation of 8-oxodG in calf thymus DNA by the PM preparations. The SRM1650 and SRM2975, with or without presence of H_2_O_2_, did not generate 8-oxodG in incubations lasting 30 minutes (figure 3A and 3B). Increasing the incubation time to 120 minutes did not show any SRM1650 or SRM2975 mediated oxidation of DNA with or without H_2_O_2 _(data not shown).

In contrast to the SRM preparations, the ASPM generated 8-oxodG in calf thymus DNA in a dose-depended manner and were more potent in the sense that it required much lower concentration of H_2_O_2 _to yield effect (10 μM versus 5 mM H_2_O_2_). Figure 3C shows concentration-dependent increases in the level of 8-oxodG after 30 minutes exposure to the 5 different samples of ASPM. No statistical analysis was made to differentiate the filters because each point represents 1–2 determinations. The results suggest that the different filters had similar ability to generate 8-oxodG and any minor differences did not appear to be associated with the small variation in iron or copper content.

### DNA damage in A549 cells

**Figure 4:**
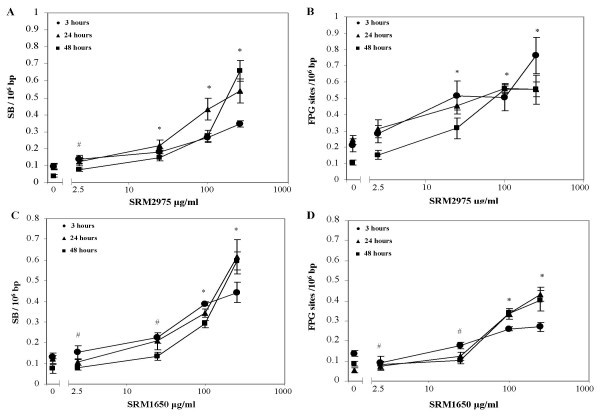
DNA damage measured by the comet assay in A549 cells exposed to SRM2975 and SRM1650 for 3, 24 or 48 hours. (A) SB, SRM2975, (B) FPG sites, SRM2975, (C) SB, SRM1650 and (D) FPG sites, SRM1650. Mean ± SEM, n = 9. * Dose where all exposure times are statistically significant compared to control (*p *< 0.01, ANOVA, except for 3 hours exposure to 100 μg/ml where SRM1650 is tested with Kruskal-Wallis test, *p *< 0.05). ^# ^Dose where a single time point is statistically significant compared to the respective control (ANOVA): (A) SRM2975, SB, 2.5 μg/ml, 48 h, *p *< 0.05, (C) SRM1650, SB, 2.5 μg/ml, 3 h, *p *< 0.05 and SRM1650, SB, 25 μg/ml, 48 h, *p *< 0.01, (D) SRM1650, FPG, 2.5 μg/ml, 3 h, *p *< 0.05 and SRM1650, FPG, 25 μg/ml, 24 h, *p *< 0.05.

Figure 4 depicts the dose-response and time curves of SB and FPG sites in A549 cells exposed to SRM1650 and SRM2975. There were significantly increased levels of SB and FPG sites after 3, 24 and 48 hours exposure to SRM2975 at doses 25, 100 and 250 μg/ml (*p *< 0.01, ANOVA). The level of SB was also significantly increased after 48 hours by SRM2975 at dose 2.5 μg/ml (*p *< 0.05, ANOVA). In cells exposed to SRM1650, the level of SB and FPG sites after 3, 24 and 48 hours were significantly increased at doses 100 and 250 μg/ml (*p *< 0.01, ANOVA, except SB at 24 hours; *p *< 0.01, Kruskal-Wallis test). The levels of SB after 48 hours and FPG sites after 24 hours were significantly increased by SRM1650 at dose 25 μg/ml (*p *< 0.01 and *p *< 0.05, respectively, ANOVA). The lowest effective SRM1650 dose in the 3 hours exposure experiments was 2.5 μg/ml for both SB and FPG sites (*p *< 0.05, ANOVA).

Table 1 shows the level of SB and FPG sites in A549 cells exposed for 24 hours to the ASPM samples. There was a general tendency of dose-dependent elevation of DNA damage, but when tested for each filter this only reached statistical significance for filter number 5 (*p *< 0.05, Kruskal-Wallis test). The level of DNA damage for all filters reached statistical significance at 100 μg/ml (*p *< 0.05, randomized block ANOVA). Due to limited amount of ASPM, we excluded the highest dose of 250 μg/ml. The exposure time of 24 hours was chosen because 3 hours exposures of SRMs had a narrow linear dose-response relationship and the cell cultures treated with SRM for 48 hours were nearly confluent.

**Table 1:** DNA damage in A549 cells exposed to authentic street particulate matter for 24 hours.

**Filter^a^**	**Dose (μg/ml)**			
	**Control**	**2.5**	**25**	**100**
	
Filter 1				
SB	0.143 ± 0.0208	0.122 ± 0.0185	0.411 ± 0.134	0.481 ± 0.149
FPG	0.069 ± 0.0452	0.066 ± 0.0286	0.205 ± 0.0690	0.191 ± 0.0488
Filter 2				
SB	0.104 ± 0.00134	0.090 ± 0.00314	0.122 ± 0.0207	0.201 ± 0.0233
FPG	0.106 ± 0.0196	0.041 ± 0.0160	0.101 ± 0.0386	0.148 ± 0.0338
Filter 3				
SB	0.116 ± 0.00947	0.097 ± 0.0160	0.352 ± 0.134	0.308 ± 0.0566
FPG	0.087 ± 0.0466	0.104 ± 0.0213	0.175 ± 0.0376	0.305 ± 0.0888
Filter 4				
SB	0.104 ± 0.0134	0.089 ± 0.0153	0.127 ± 0.0110	0.453 ± 0.178
FPG	0.106 ± 0.0196	0.076 ± 0.0253	0.102 ± 0.0177	0.210 ± 0.0522
Filter 5				
SB	0.150 ± 0.00919	0.197 ± 0.0415	0.218 ± 0.0395	0.399 ± 0.0309^b^
FPG	0.289 ± 0.0266	0.513 ± 0.0552	0.415 ± 0.0584	0.621 ± 0.0276^b^
All filters				
SB	0.123 ± 0.00975	0.119 ± 0.0204	0.246 ± 0.0586	0.368 ± 0.0512^c^
FPG	0.131 ± 0.0400	0.160 ± 0.0888	0.200 ± 0.0575	0.295 ± 0.0853^c^

^a ^The data from filter 1–5 represent lesions/10^6 ^bp as mean ± SEM, n = 6.

^b ^Statistical significant compared to the control, *p *< 0.05 (Kruskal-Wallis test), N_total _= 24.

^c ^Statistical significant compared to the control, *p *< 0.05 (randomized block ANOVA), the statistical analysis was based on the mean value of each filter (N_total _= 20).

**Figure 5:**
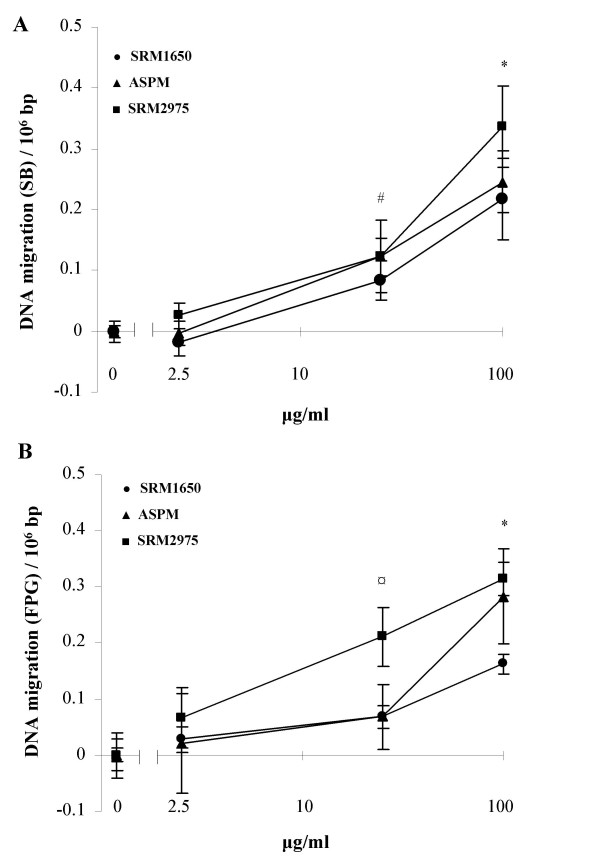
Baseline-adjusted SB (A) and FPG sites (B) in A549 cell cultures exposed 24 hours to SRM1650, SRM2975 and ASPM. The data are obtained from figure 4 and table 1. Data points are mean ± SEM (SRMs, n = 9; ASPM, n = 6). There was no significant difference between the particle's ability to induce SB and FPG sites at any doses (*p *> 0.05, Kruskal-Wallis test).

Figure 5 shows the level SB and FPG sites generated by ASPM (all filters), SRM1650 and SRM2975 after 24 hours exposure. For the purpose of comparing the genotoxicity of ASPM and SRMs, the data from figure 4 (250 μg/ml is excluded) and table 1 were baseline adjusted. Comparisons of the effect at doses 25 and 100 μg/ml did not reveal significant differences in SB and FPG sites between the types of PM preparations (*p *> 0.05, Kruskal-Wallis test).

## Discussion

In this study we have shown that there are no clear differences in the genotoxicity of ASPM and the SRM1650 and SRM2975 preparations assessed as the dose-response relationship at different periods of incubation in A549 cells. This genotoxicity cannot be predicted from the ability of the PM to induce cytotoxicity, where mainly SRM1650 had effect, or oxidative damage to DNA in a cell-free environment, where only ASPM had effect.

In the present study, ASPM generated high levels of 8-oxodG in calf thymus DNA, whereas SRM1650 and SRM2975 was not able to oxidize DNA at even high doses of H_2_O_2_. These data are consistent with previous studies on authentic urban particles showing increased 8-oxodG in the presence of H_2_O_2 _[26,27]. However, samples of total suspended street particulates obtained at different locations of Maastricht did not generate 8-oxodG in Salmon testis DNA in a manner that reflected the variation in the local traffic intensity [28]. Since the oxidation of DNA in a cell free environment containing H_2_O_2 _is likely to be caused by metal-catalyzed reactions in the solvent, it is possible that the lack of oxidizing effect of the SRM1650 and SRM2975 is because the levels of transition metals are very low and/or not available to facilitate catalysis. E.g. the concentration of iron in the ASPM samples was 1800 times higher than for the SRMs (37 versus 0.02 μg/mg, respectively), although the small variation between the filters was not reflected in the small variation in DNA oxidation. It has been shown that iron and iron-containing mineral fibres dose-dependently can generate 8-oxodG in naked DNA [29,30]. In addition, PM collected in an urban location in Washington DC by NIST (SRM1649) also generated 8-oxodG in isolated DNA [31]; this preparation has higher concentration of transition metals than both SRM1650 and SRM2975 [22]. Similarly, urban air PM induced 8-oxodG in DNA measured by immunochemical methods with some differences between different size fractions [26,27]. Authentic preparations of exhaust particles from light duty diesel engines without reported elemental composition could oxidize isolated DNA at very high doses from 5 mg to 20 mg of DEP without H_2_O_2 _or 10 mg/ml with H_2_O_2 _[32,33]. Collectively, these investigations indicate that DEP, as compared to urban air PM, possess low direct oxidizing ability toward DNA in cell free environments unless at very high concentrations. In addition to transition metals, ASPMs might also contain larger quantities of semiquinone radicals that can generation of 8-oxodG in calf thymus DNA by direct oxidations of guanine bases [34].

Contrary to the cell free system, the PM samples were able to induce SB and FPG sites in the DNA of A549 cells. The level of SB elicited by ASPM was similar to another recent and independent investigation of the PM collected on Jagtvej in 2005; that report indicated that exposure to 25 μg/ml for 24 hours increased the DNA migration from 7% to 14% [25]. This is the same as a net increase of 0.1 SB/10^6 ^bp, using the conversion factor from The European Standards Committee on Oxidative DNA damage (assuming that 1% corresponds to 0.0185 SB/10^6 ^bp). In our study, the net increase of SB at the same dose and exposure duration was 0.083, 0.12, and 0.12 SB/10^6 ^bp for the SRM1650, SRM2975, and ASPM (all filters), respectively. Elevated levels of SB have been reported for a variety of authentic urban particles and SRMs in various cell types [35]. E.g. exposure of A549 cells to street particles from Duisburg (Germany) and Stockholm (Sweden) increased the levels of SB at a dose of 20 μg/cm^2 ^[27,36], which is close to threshold of effect in our study (20 μg/cm^2 ^corresponds to 38 μg/ml in our experimental setup). Our investigation extends the information of genotoxicity to encompass the oxidized guanines, including the pre-mutagenic 8-oxodG lesions. The net increase in FPG sites was somewhat higher in cell cultures exposed to 25 μg/ml for 24 hours of SRM2975 (0.21 lesions/10^6^bp) than for the SRM1650 and ASPM samples (approximately 0.068 lesions/10^6 ^bp; figure 5b). Importantly, the lowest effective dose generating oxidative damage to DNA was not associated with altered viability or reduced colony forming ability. This indicates that the genotoxicity is caused directly by the presence of the particles and it is not a secondary effect due to cytotoxicity and altered replication. The induction of oxidative damage to DNA by the SRM preparations is unlikely to be secondary to inflammatory responses elicited in the A549 cells. We have previously reported that cytokine responses required exposure to 500 μg/ml of SRM1650 for 2 hours, whereas the level of SB was significantly increased after 2 hours with only 100 μg/ml [17]. This is also in line with findings of PM-induced DNA damage in a variety of cell types, including fibroblasts, hepatocytes and intestinal epithelium cell lines [35].

The measurement of DEP-induced FPG sites has not been investigated before in lung epithelial cells exposed to authentic street particles, whereas two studies have reported elevated levels of 8-oxodG in lung epithelial cells exposed to urban PM, detected by immunohistochemistry and HPLC-EC, but only at one dose [26,31]. Investigations on oxidized guanines are probably more relevant for carcinogenesis than the types of DNA damage detected as SB by the comet assay. In addition, we have observed that exposure to air pollution elicits more pronounced effect in terms of FPG sites compared to SB in mononuclear blood cells of humans in Copenhagen and Cotonou, which is the heavily air polluted capital of the Republic of Benin [5-7]. In this respect, the dose-dependent increase in FPG sites observed in this in vitro study provides firm evidence for the induction of oxidative damage to DNA by air pollution PM. It also outlines that the SRM1650 and SRM2975, which are samples of DEP, are reliable surrogate PM for authentic air pollution particles when measuring DNA damage in terms of SB an FPG sites in the comet assay.

In conclusion, we show that ASPM and SRMs differ in their ability to form 8-oxodG in isolated DNA, whereas they induce SB and FPG sites in A549 cells in a dose-dependent manner. The SRM1650 preparation generated cytotoxicity at lower dose than the SRM2975 and ASPM samples, but it was not associated with differences in the genotoxic potency. In general, elevated cellular level of DNA damage was detected at doses with no overt cytotoxicity. We conclude that SRM1650 and SRM2975, which are well-characterized samples of DEP, are good model particles for ASPM, due to their equally DNA damaging effects in lung cells.

## Methods

### Samples of authentic street PM and SRMs

Samples of SRM1650 and SRM2975 were obtained from the National Institute of Standards and Technology (Gaitersburg, MD, USA). The SRM1650 and SRM2975 are samples of diesel PM collected from the engine of heat exchangers and filtering system of an industrial diesel-powered forklift, respectively [20,21]. ASPM was collected on Millipore type RA membrane filters, consisting of a gridded membrane filter made from mixed esters of cellulose, as total suspended particulates at a monitoring station on Jagtvej in Copenhagen on five weekdays in September 2000. During this period the annual TSP concentration was <50 μg/m^3 ^at Jagtvej. Based on measurements from the PM10 fraction was has been estimated that 11 μg/m^3 ^of PM_10 _concentration originated from traffic and the rest (22 μg/m^3^) derive from road dust, tire wear, road salt and long-range transport of particles. Approximately 75% and 25% of the traffic-generated PM_10 _originated from diesel and petrol powered vehicles, respectively [37]. The particles were collected and analyzed as a part of the Danish Air Quality Monitoring Programme (conducted by the Danish National Environmental Research Institute) and stored in dark containers at room temperature. The collection volume was 60 m^3 ^and the total collected material was 56.5 ± 13.2 μg/cm^3 ^(mean ± SD, n = 5). The elemental composition (Al, Si, S, Cl, K, Ca, Ti, V, Cr, Mn, Fe, Ni, Cu, Zn, Ga, As, Se, Br, Rb, Sr, Zr, Mo, Sn, Sb, Ba and Pb) was measured by proton induced X-ray emission analysis at the National Environmental Research Institute, Denmark [38]. Each filter was placed in a vial with 5 ml of ultrapure water and subjected to ultrasonication for 5 min at room temperature to liberate particles. Stock suspensions of these ASPM samples were stored at -20°C until used in experiments. The concentration of the ASPM stock solutions (μg/ml) was calculated from the total concentrations on the filters, assuming that all material was liberated from the filters. The size distribution was not defined. Stock suspensions with a concentration of 2500 μg/ml of SRM1650 and SRM2975 were prepared in ultrapure water. The suspensions were ultrasonicated and vortexed prior to dilution with ultrapure water or cell medium to the final concentrations.

### Cell culture

A549 cells (American Type Culture Collection, Manassas, VA, USA), kindly provided by the National Research Centre for the Working Environment, Denmark, were grown in F12 nutrient mixture (HAM) supplemented with 10% heat inactivated foetal bovine serum, 1% L-glutamine and 1% penicillin-streptomycin (incubation at 37°C, 5% CO_2_). All were Gibco^® ^cell culture products purchased from Invitrogen™, Denmark.

Plates of different sizes have been used in the experiments of LDH, colony forming ability, and comet assay. Exposures are reported as concentrations (μg/ml). For comparison between the experiments in terms of cell area, the highest concentration (250 μg/ml) in the 24 hour incubations corresponds to 132 μg/cm^2 ^(comet assay), 78 μg/cm^2 ^(colony forming assay), and 166 μg/cm^2 ^(LDH assay).

### LDH assay

The cytotoxicity of SRMs and ASPM was measured as LDH activity in cell medium by the Cytotoxicity Detection Kit from Roche Applied Science, Penzberg, Germany. An increase in the number of dead or cell membrane-damaged cells increases the LDH activity in the cell culture supernatant. In this assay the well area was 0.3 cm^2 ^(96 well culture plates) and the recommended amount of solution was 200 μl in each well.

### Colony forming assay

The ability of a single cell to form a colony of cells after exposure to SRMs and ASPM was determined by the colony forming assay as described previously [39]. 3 × 10^5 ^cells were seeded into 6 well culture plates (9.6 cm^2^/well) in 3 ml cell culture medium. After 48 hours of incubation, cells were washed with phosphate buffer saline, and 3 ml SRM1650, SRM2975 or ASPM samples with concentrations corresponding to doses at 0, 2.5, 25, 100 and 250 μg/ml was added. In this assay, suspensions of ASPM were pooled from different filters, because of limited amount of material. Hereafter 200 cells were seeded into 6 well culture plates with 3 ml medium and incubated for 5 days in 5% CO_2 _and 37°C. The cells were treated in methanol for 10 minutes and stained with trypan blue for 30 minutes. The number of colonies with more than 50 cells was counted using a magnifying glass.

### Measurement of DNA damage in A549 cells by the comet assay

DNA damage was measured as the formation of SB and FPG sites in the DNA by the comet assay as previously described [15,40]. For experiments, 10^5 ^cells were seeded into 24 well culture plates (1.9 cm^2^/well), and grown for 48 hours (~80% confluence). The cells were treated with 1 ml SRM stock suspension diluted with cell culture medium to final concentrations; 0, 2.5, 25, 100 and 250 μg/ml or with 1 ml ASPM solution diluted with cell culture medium to final concentrations; 0, 2.5, 25, and 100 μg/ml. The cells were harvested after 3, 24 or 48 hours exposure with trypsin-EDTA (Invitrogen™, Denmark) and finally centrifuged at 3000 rpm for 6 minutes before addition of agarose gel.

The cell culture experiments were carried out on three different days for both SRMs and ASPM. Cells were exposed to the SRMs and ASPM in triplicates/day (N_total _= 9) and duplicates/day (N_total _= 6), respectively.

The samples were coded before visual scoring, where the level of SB and FPG sites was obtained by scoring 100 nuclei using a five-class scoring system (arbitrary score range: 0–400). The primary comet assay endpoints in arbitrary units were transformed into lesions/10^6 ^base pair (bp), using an investigator-specific X-ray calibration curve and assuming that human diploid cells contain 4 × 10^12 ^Dalton DNA (corresponding to 6 × 10^9 ^bp) as reported previously [41].

### Measurement of 8-oxodG in calf thymus DNA

Different concentrations of ASPM were added to solutions of calf thymus DNA (150 μg/ml) in a total volume of 0.5 ml with 10 μM of H_2_O_2 _and incubated for 30 min at 37°C. For the determination of 8-oxodG generation by SRM1650 and SRM2975, SRM preparations were added to solutions of calf thymus DNA (300 μg/ml) in a total volume of 1 ml with or without 5 mM of H_2_O_2 _and incubated for 30 or 120 minutes at 37°C. The exposed DNA was precipitated, subjected to enzyme digestion and high performance liquid chromatography analysis with electrochemical detection (HPLC-EC) as described previously [42]. The results are presented as the ratio between 8-oxodG and the normal dG.

### Statistics

All variables were tested for normal distribution using the Shapiro-Wilks test. If groups did not show homogeneity of variance by Levene's test, they were either log-transformed or cubic root transformed before further statistical analysis. Data that did not show homogeneity variance were analysed by the non-parametric Kruskal-Wallis test, with post-hoc Tukey-type multiple comparison test or the Mann-Whitney U test. Data that had homogeneity of variance were tested with ANOVA with post-hoc Fisher test. The dose-response relationship in DNA damage detected by the comet assay of ASPM samples was tested by a randomized block ANOVA analysis; only the mean values for each treatment was included in the test (corresponding to N = 20). The data on comet assay endpoints displayed inter-experiment variation that made it difficult to compare different samples directly. Therefore, the raw data are baseline-adjusted; the mean of the zero dose culture was subtracted from each data point. All variables were tested at the 5% probability significance level. P-values refer to post-hoc tests. The statistical analysis was performed in Statistica 5.5 (StatSoft, Inc., Tulsa, USA).

## List of abbreviations

8-oxo-7,8-dihydro-2'-deoxyguanosine; ASPM: Authentic street particulate matter; bp: Base pair; DEP: Diesel exhaust particles; FPG: Formamidopyrimidine glycosylase; HPLC-EC: High performance liquid chromatography with electrochemical detection; LDH: Lactate dehydrogenase; NIST: National Institute of Standards and Technology; PAH: Polycyclic aromatic hydrocarbons; PM: Particulate matter; SB: Strand breaks; SRM: Standard reference material.

## Competing interests

The authors declare that they have no competing interests.

## Authors' contributions

SL and PM conceived the study. PHD, SL and PM designed experiments. PHD made all experiments, except the data on 8-oxodG is calf thymus DNA that was analyzed by a laboratory technician under the supervision of SL and PM. The statistical analysis was carried out by PHD, who also provided the first draft of the manuscript. SL and PM have revised the draft critically and have approved of the final version to be published. All authors have read and approved the final manuscript.
